# Dietary Diversity and Prostate Cancer in a Spanish Adult Population: CAPLIFE Study

**DOI:** 10.3390/nu12061694

**Published:** 2020-06-06

**Authors:** Naomi Cano-Ibáñez, Rocío Barrios-Rodríguez, Macarena Lozano-Lorca, Fernando Vázquez-Alonso, Miguel Arrabal-Martín, José Matías Triviño-Juárez, Inmaculada Salcedo-Bellido, José Juan Jiménez-Moleón, Rocío Olmedo-Requena

**Affiliations:** 1 Department of Preventive Medicine and Public Health, University of Granada, 18016 Granada, Spain; ncaiba@ugr.es (N.C.-I.); macarenalozano@ugr.es (M.L.-L.); isalcedo@ugr.es (I.S.-B.); jjmoleon@ugr.es (J.J.J.-M.); 2 Consortium for Biomedical Research in Epidemiology and Public Health (CIBERESP), 28029 Madrid, Spain; 3 Instituto de Investigación Biosanitaria (ibs.GRANADA), 18014 Granada, Spain; arrabalm8@gmail.com; 4 Urology Department, Virgen de las Nieves University Hospital, 18014 Granada, Spain; fvazquezalonso@gmail.com; 5 Urology Department, San Cecilio University Hospital, 18016 Granada, Spain; 6 Primary Care Center Zaidin Sur, Granada Metropolitan Sanitary District, 18007 Granada, Spain; jmtjuarez@hotmail.com

**Keywords:** prostate cancer, dietary diversity, nutrient adequacy, CAPLIFE study, case-control study

## Abstract

Dietary diversity (DD) is a key component of a high-quality diet, providing the adequate nutrient requirements. However, the role of DD on prostate cancer (PCa) is still uncertain. The aim of this study was to evaluate the relationship between DD, adequate nutrient intake and PCa, according to the aggressiveness of the tumor. The CAPLIFE (CAP: prostate cancer; LIFE: lifestyles) study is a population-based case-control study including a total of 402 incident PCa cases and 302 controls. The DD score (DDS), adjusted by total energy intake, was collected through a validated food frequency questionnaire. Nutrient adequacy was defined according to European Dietary Recommendation Intake for men. The aggressiveness of PCa was determined according to the International Society of Urology Pathology classification. The association between DDS, nutrient intake and PCa was assessed by logistic regression models with adjustment for potential confounding factors. DDS was similar for PCa cases and controls, independent of PCa aggressiveness. According to each food group DDS, the protein group showed the highest mean score in all the subgroups analyzed. However, no differences were observed for each of the DDS components. The DDS, the variety of the group’s food intake, and the adequate nutrient intake, were not associated with PCa.

## 1. Introduction

Prostate cancer (PCa) has increased in recent years and become widespread worldwide [1]; with around 450,000 estimated new cases per year [2]. In Spain, the incidence rate of PCa has been increasing rapidly and is now the most frequently diagnosed male cancer [3]. Moreover, the disease is considered a significant public health burden and a major cause of morbidity and mortality among males, with increasing healthcare resource utilization and cost [4].

Advanced age, black race, a family history of PCa, and specific genetic polymorphisms have been identified as risk factors for PCa [5]. None of these factors can be considered potentially modifiable. On the other hand, these factors cannot explain by themselves the existing geographic differences for PCa and the continuing increase in tumor rates over recent years. Thus, the epidemiology of PCa suggests that it is a multifactorial disease. Environmental factors and personal lifestyles could be implied in its etiology, promoting targets for the prevention of PCa [6].

Regarding diet, accumulating evidence suggests that diet is associated with the development of some cancers, for example, breast and colorectal cancer [7,8]. Nevertheless, the role of diet in PCa remains unclear [9]. The third guidelines proposed by the World Cancer Research Fund/American Institute for Cancer Research (WCRF/AICR), in 2018, suggests that there is limited evidence for this issue [10]. To date, the evaluation of the role of our diet and the risk of developing PCa has been conducted through isolated nutrients, food group’s intake or specific dietary patterns.

In terms of nutrient intake, while some authors have not found any association between any particular intake of nutrients and PCa [9,11], other studies have pointed out that an insufficient intake of micronutrients is related to an increase in the risk of PCa [12,13,14]. For example, the deficit of vitamins A and D has been linked to malignant prostatic epithelial cell differentiation [15]; and a deficit of some minerals, specifically zinc and calcium, has been associated with an increased risk of PCa [16]. Along the same lines, while some epidemiological studies observed a protective relationship for fruits and vegetables, such as cruciferous vegetables [17] and tomatoes [18] and PCa, other studies did not find any relationship [19]. Other studies that focused on the adherence to overall dietary patterns, such as a Mediterranean Diet, have reported that the greater adherence to this dietary pattern was associated not only with a reduction in PCa risk [20] but also with lower rates of mortality [21]. In addition, differences in associations were observed in these previous studies depending on tumor aggressiveness. This supports the idea that an epidemiological analysis should be conducted, stratifying the population according to these criteria [22].

The inconsistencies found in the relationship between PCa and singles nutrients or food factors could be because the effect of specific dietary components does not explain the complex combinations of nutrients that are likely to have interactive or synergistic effects [23]. The dietary diversity score (DDS) is a simple score that assesses the variety of a selected number of food groups (i.e., dairy, proteins, fruits, vegetables, and grains) [23]. The DDS can be considered a global indicator of the quality of the diet and its ability to meet our nutrient needs [24]. Furthermore, according to the current dietary guidelines, an adequate dietary pattern should include a wide variety of food, in order to meet micronutrient requirements in adults, through a diverse and nutrient-dense diet [25,26], factors included as part of DDS. The relationship between DDS and PCa has not been explored previously, and it may be an opportunity to identify possible modifiable risk factors to prevent PCa. Therefore, the aim of the present study was to evaluate the relationship between dietary diversity and adequate nutrient intake with the risk of PCa, according to the grade of aggressiveness of the tumor.

## 2. Materials and Methods

### 2.1. Study Design and Setting

The CAPLIFE (CAP: prostate cancer; LIFE: lifestyles) study, a population-based case-control study, was developed to evaluate the association between lifestyles and PCa. Participants were enrolled from May 2017 to December 2019. Cases were selected from the two main university hospitals in the province of Granada (Andalusia, Spain): Hospital Universitario Virgen de las Nieves and Hospital Universitario San Cecilio PTS.

The study was conducted according to the guidelines set in the Declaration of Helsinki and all the procedures involving human subjects were approved by the Ethics Committee of Biomedical Research of Andalusia, March 2017. All the men included in the study were fully informed about the study objectives and signed informed consent before their voluntary participation. The patients were informed that it was possible to withdraw their consent at any time, without prejudice to his treatment or the relationship with his doctor. Confidentiality of data was secured, removing any personal identifiers in the dataset.

### 2.2. Participants

**Figure 1:**
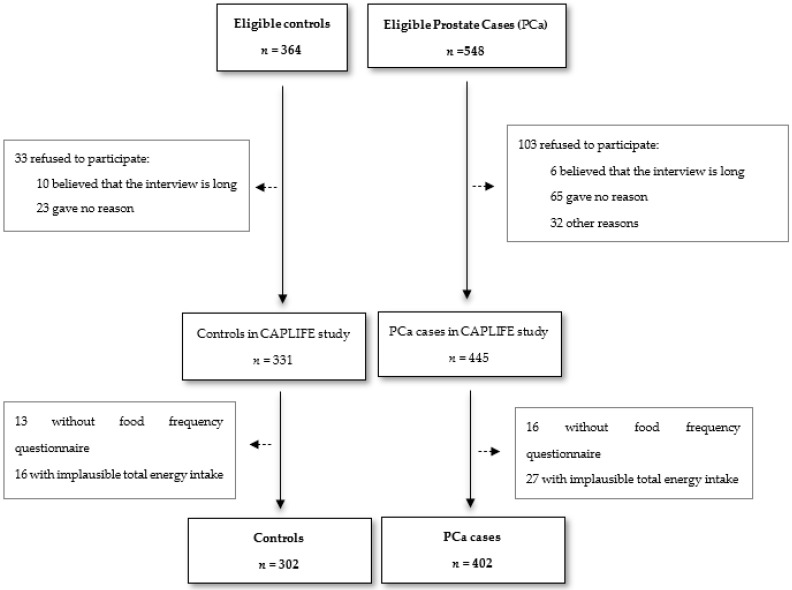
Flow-chart CAPLIFE (CAP: prostate cancer; LIFE: lifestyles) study.

The eligible cases were those subjects who met the following selection criteria: (1) new diagnosis of PCa with histological confirmation (according to the International Classification of Diseases 10th Revision (ICD-10): C61) [27]; (2) age between 40 and 80 years; and (3) residence in the coverage area of the reference hospitals during at least 6 months before their recruitment. The same selection criteria were used for controls except for the diagnosis of PCa. The incident cases were selected from the Urology Department of the participating hospitals, using the Pathological Anatomy listings and checking the new positive biopsies for PCa. When a new diagnosis of PCa occurred, the subject was informed about the study by his urologist and invited to participate in the study. The controls were randomly selected from the population lists assigned to general practitioners of the primary care centers located in the catchment area of the hospitals where the cases were selected (Granada-Metropolitan Sanitary District). The controls were matched to cases by age, with a maximum age difference of 5 years, based on information from the Granada Cancer Registry: a population-based cancer registry with data quality certified by the International Agency for Research on Cancer (https://www.registrocancergranada.es/es/). After applying the selection criteria, a sample of 402 incident PCa cases and 302 controls was assessed (Figure 1). After the cases and controls signed informed consent, they completed an epidemiological questionnaire specifically developed for this study. The interview was carried out by trained interviewers.

### 2.3. Data Sources and Variables

Face-to-face interviews and patients’ medical records were used to collect the information of interest. The patients with PCa were interviewed before starting any type of treatment for the disease, and therefore all the collected information always referred to the period prior to the diagnosis of PCa. The personal interview with the participants allowed the collection of information on the following: (1) sociodemographic and anthropometric characteristics, including age, education level, marital status, work occupation, and body mass index (BMI), one year before diagnosis, with self-reported height and weight; and (2) lifestyle habits, comprising smoking status, alcohol consumption, physical activity (PA), and information about diet during the last 12 months. The PA information was collected through the International Physical Activity Questionnaire (IPAQ), validated for the Spanish population [28]. Using the IPAQ information, three categories were created to measure the level of physical activity for each participant according to the following standards: (1) a high level of PA (PA levels equate to approximately one hour per day or more of moderate or high-intensity physical activity); (2) moderate level of PA (half an hour of PA or more of moderate-intensity PA on most of the days); and (3) low level of PA (not meeting any of the criteria for either moderate or high levels of PA).

#### 2.3.1. Dietary Assessment

To gather the dietary information, a 134-item semi-quantitative food frequency questionnaire (FFQ), previously validated for the Spanish population, was used [29,30]. The dietary information referred to the 12 months before the interview. The FFQ is based on the frequency of consumption of different foods and the portion size for each of the included foods. The indicated frequencies of consumption were converted to intake per day and multiplied by the weight of the standard serving size in order to estimate the intake in grams per day. Nutrient information was derived from Spanish food composition tables [31,32]. Subjects with an intake of less than 800 kcal/day and more than 4000 kcal/day were not included in the analysis as these were considered as implausible extreme energy intakes [33].

#### 2.3.2. Dietary Diversity Score Construction

The dietary diversity score (DDS) was calculated using the method originally developed by Kant et al. [23], which has recently been used by Farhangi et al. [34] and Cano-Ibáñez et al. [26,35]. Five food groups were considered in the construction of the DDS: vegetables, fruits, cereals, dairy products, and the protein food group. The selection of these five groups was based on the food groups recommended by the Spanish guidelines’ nutritional pyramid [25]. Table 1 shows a detailed description of the food groups, and the subgroups considered in the DDS and the consumption recommended in servings per/day, as well as the specific foods included in each group. Non-recommended food groups [36], including sugar food groups (pastries, pies, biscuits, chocolate, fruit in sugar syrup and fruit juices) and food groups with high contents in salt or saturated fat (butter, margarine, unhealthy vegetable fats, red meat, processed meats, sauces, pre-cooked dishes, condiments and snacks) were not included in the analysis as they are well known unhealthy products, and their variety is not desirable [37].

According to the recommendations of the Spanish nutritional pyramid [25], optimal dietary diversity should be considered if a subject consumes at least half of the recommended servings per day for each of the items that make up the food group, scoring with 2 points for each item. For example, if the Spanish nutritional recommendations advise a usual fruit intake of 3 servings per day, then a subject should consume at least 1.5 servings/day for each fruit item. Thus, if the consumption per day for a fruit item is lower than 1.5 servings, the value for this item will be 0; conversely, if the consumption is higher than 1.5 servings, the value will be 2. For the five considered groups, the procedure is similar. To equalize the weight of each food group, all the scores were rescaled on a scale between 0 and 2. To do this, we added the value for each item, this value is multiplied by 2, and then the result is divided by the maximum score in the food subgroup assessed. Therefore, continuing with the example of the fruit group, if a subject has an intake of citrus fruits and other seasonal fruits higher than 1.5 servings/day for each item and lower than 1.5 servings for tropical fruits then the value for fruits will be equal to 4 (2 + 2 + 0), and the rescaled value will be 4 multiplied by 2 and divided by 6 (maximum score for fruits). Thus, the score of each of the 5 predefined food groups ranged from 0 to 2 points. The total DDS is the sum of the scores of the five main groups, theoretically ranging between 0 (minimum) and 10 points (maximum).

The score was adjusted for the total energy intake using the residual method as recommended by Willet et al. [33], independently for the case and control group, due to the general concern that high food variety might be a consequence of the overconsumption of energy [38]. Energy-adjusted DDS was stratified into quartiles (Q), establishing cut-off points based on the distribution of controls. The DDS cut-off points were Q1: 4.7, Q2: 5.7, Q3: 6.6, and Q4: 8.5 points. On the other hand, the variety within each food group was classified into four categories (C) of diversity intake, defined a priori, which have been recently used in the literature [26,39]: C1 = 0 points, C2 > 0 to < 0.5 points, C3 ≥ 0.5 to < 1 point and C4 ≥ 1 point for vegetable and cereal groups; C1 = 0 points, C2 > 0 to < 1 point, C3 ≥ 1 to < 1.5 points and C4 ≥ 1.5 points for protein, fruit, and dairy products groups.

#### 2.3.3. Nutrient Adequate Intake

Vitamins A, B_6_, B_9_, B_12_, C, D, E, as well as minerals such as calcium, magnesium, iodine, potassium, selenium, and dietary fiber were analyzed. For each nutrient, the individual daily intake was compared with the recommended intakes according to the requirements established by the European Food Safety Agency (EFSA) for men [39]. Taking into account the average requirements (AR), average intake (AI), and upper level intake (UL), 3 categories were built: (i) deficient intake (intake below AR/AI); (ii) adequate intake (intake between AR/AI and UL); and (iii) excessive intake (intake higher UL). To decrease the potential measurement errors derived from the use of the FFQ (overestimation bias), we calculated the proportion of individuals with intakes below two-thirds (2/3) of the Dietary Reference Intakes (DRIs) of the EFSA criteria, to define a deficient intake, as other authors have reported previously [40]. The intake of vitamin and other trace element supplements were excluded.

### 2.4. Measurement of the Tumor Aggressiveness

The tumor Gleason score was obtained from the medical history. Prostate cancer aggressiveness was established using the classification of the International Society of Urological Pathology (ISUP) [41]. According to ISUP classification, two categories of aggressiveness were defined: low aggressiveness [ISUP 1 (Gleason 3 + 3) and ISUP 2 (Gleason 3 + 4)] and high aggressiveness [ISUP 3 (Gleason 4 + 3), ISUP 4 (Gleason 4 + 4, Gleason 3 + 5, Gleason 5 + 3), ISUP 5 (Gleason 5 + 4, Gleason 4 + 5, Gleason 5 + 5)] [42].

### 2.5. Statistical Analysis

Qualitative variables were analyzed through the frequency distribution, whereas quantitative variables were expressed as means and standard deviation (SD). Pearson chi-square test and the Student’s t test were used to assess the differences in the characteristics and adequate nutrient intake between the PCa cases and controls. Logistic regression models were performed to calculate crude odds ratios (cOR) and adjusted odds ratios (aOR) with the corresponding 95% confidence intervals (95% CI). These models were used to evaluate the relationship between the risk of PCa according to the total DDS, food group variety, and the micronutrient intakes as the main independent variables. Odds ratios and their 95% CI were calculated considering the lowest quartile/category (the least recommendable) as the reference. To control for potential confounding factors, the results were adjusted for age, BMI, educational level, PA level, smoking status, alcohol intake, and first-degree family history of PCa. Finally, we performed stratified analyses by aggressiveness: low aggressiveness (ISUP 1, 2) and high aggressiveness (ISUP 3, 4, 5) in order to analyze its role in the evaluated relationship. Statistical significance was set at *p* < 0.05. Statistical analysis was performed using Stata (15.0, StataCorp LP, Texas, TX, USA).

## 3. Results

### 3.1. Characteristics of CAPLIFE Study Participants

Table 2 shows the comparison of the sociodemographic, anthropometric and lifestyle variables for both groups, PCa cases and controls. The cases and controls were similar for all of the variables except for age; 67.8 years old (SD: 7.4) for the PCa group vs. 65.3 (SD: 8.2) for the controls. More than three-quarters of the PCa cases were cases of low aggressiveness (76.9%); ISUP 1 or 2.

### 3.2. DDS and Variety in Each Food Group and Risk of PCa

The description of DDS and its components for controls, overall PCa cases, and stratifying by aggressiveness is shown in Table 3. Global DDS was similar for PCa cases and controls, independent of the PCa aggressiveness. According to each food group DDS, the protein group showed the highest mean score in all the subgroups analyzed. However, no differences were observed for each of the DDS components either. When the relationship between the DDS and the PCa is analyzed, considering the potential confounding factors (Table 4), no relationship was observed for the global DDS nor for each food group DDS.

### 3.3. Prevalence of Participants with Adequate, Deficient, or Excessive Nutrient Intake

Table 5 shows the prevalence of participants with an adequate, deficient, or excessive nutrient intake. Participants with a low aggressiveness PCa showed a higher prevalence of adequate selenium intake than the participants in the control group (97.8% vs. 93.7%; *p* = 0.030).

### 3.4. Relationship between Nutrient Adequate Intake and PCa, According to Tumor Aggressiveness

The multivariate adjusted odds ratios (aOR) for the risk of PCa and each micronutrient intake are presented in Table 6. After adjustment for potential confounders, the odds of developing PCa was 67% lower for participants with an inadequate intake of selenium compared to those with an adequate intake. Nevertheless, when we take into account the stratification of selenium intake according to adequate, deficient and excessive intake our results showed a non-significant association—deficient intake aOR = 0.45 (95% CI 0.16–1.22) and excessive intake aOR = 0.12 (95% CI 0.01–1.03)—(data not shown).

## 4. Discussion

In this case-control study, the relationship between the DDS and adequate nutrient intake and their association with PCa was evaluated. The findings suggest that diet, specifically the diversity of the dietary pattern and adequate specific nutrient intake, were not associated with PCa overall, nor when considering tumor aggressiveness.

The relationship between food intake and the risk of PCa has been largely examined in several studies from the mid-1990s to the present day. Diet, regardless of how it is measured, has not been definitively associated with PCa, and to date, published results are highly inconsistent. Some studies have suggested that the intake of specific food groups such 1 as legumes [43], yellow-orange and cruciferous vegetables [44], and tomato sauce [45] would be associated with PCa in a protective way. Meanwhile, the intake of dairy products, meat, and saturated fat of animal origin have been associated with the risk of PCa [46,47]. However, nowadays, it is considered more recommendable to measure the overall dietary pattern. Therefore, the interest in individual food or nutrients as potential factors associated with PCa has decreased substantially in recent decades. The study of the overall dietary pattern instead of the traditional approach is more intuitive and objective [24]. Accordingly, we used a DDS originally developed by Kant et al. [23] that reflects the diversity of food intake, and until now has not been used in the assessment of PCa risk. The basis of our hypothesis was that the diversity of the diet can be considered a good indicator of its quality and, therefore, the higher the quality of our diet, the greater the health benefit.

This study showed that neither the total DDS nor the variety within each of the food groups were related to a lower risk of PCa; contrary to our hypothesis. Some reasons could explain these results: (i) it could be that there really is no association between diet and PCa; (ii) DDS may not be ideal to identify differences between groups. When there are no defined limits to classify the population according to DDS, and the quartiles obtained from the control group are selected to analyze its effects, this index becomes dependent on the population being studied. Hence, taking into account that our participants are mostly elderly, and that the evidence suggests that diet quality increases in old age [48,49], the quality of the diet could be similar in the whole sample making it difficult to find any association; and (iii) another reason that could explain the lack of association could be that the evaluation of the diet is complex. Despite having used a validated tool for the FFQ [50], the analyzed outcome (PCa) was not classically related to dietary intake [51]. Therefore, in the case of bias (measurement bias), it would be a non-differential classification bias. As no previous study has researched the effect of diet diversity (DD) intake on PCa, we cannot directly compare our results with other epidemiological studies. Additional prospective studies are needed to verify if any relationship between DD and PCa risk could exist.

On the other hand, as indicated in the third AICR report generated in 2018 [10], there is still limited evidence between the nutrient intake and the increased risk of PCa. The nutritional content of the diet and the relationship between the nutrients and PCa risk is still unclear [52]. Specifically, with respect to selenium, there are studies that find a link with the PCa, pointing out an anti-inflammatory effect of supplemental selenium as a possible causal pathway [53,54]. Similarly, the Nutritional Prevention of Cancer Trial, originally developed to study the effect of selenium on non-melanoma skin cancer, found a 63% reduction in PCa risk among men taking selenium supplements [55]. Conversely, there are studies that have not found an association between selenium and prostate cancer [56,57]. Our results showed that an inadequate intake of this nutrient was significantly related to a lower risk of PCa. We cannot rule out the possibility that this finding was a false positive conditioned by the study power [58]. Moreover, when the categories were analyzed separately (deficit vs. normal vs. excess), it seems the observed protective association is due mainly to the highest levels of selenium. However, the results did not reach the statistical significance, and are consistent with the IARC.

The present study has some limitations. First, although the semi-quantitative FFQ has been validated for the Spanish population [29], self-reporting questionnaires and memory problems could lead to information bias. However, in that case, a non-differential misclassification bias would occur, so estimations would tend toward the null. Moreover, to correct possible errors in this method, we excluded participants with energy intakes outside of the predefined limits [33], and we used the residual method to adjust the DDS for energy intake. Second, although this tool specifies the usual portion size as part of the question on frequency, it may not be the perfect instrument for measuring micronutrient intake. Hence, to overcome this possible bias owing to the nature of the FFQ, we judged an adequate intake to be only when the intake reached two-thirds of the DRIs and assumed that the inadequate micronutrient intake (deficient or excessive intake) will be higher than the estimated figures [39]. Lastly, we estimated a DDS using the methodology developed by Kant et al. [23]. This DDS excludes non-recommended food products that are high in sugar (e.g., sweets, snacks, juices and sweet beverages), saturated lipids, and meats owing to the high-energy density of these foods, as well as their low-nutrient density with high levels of sodium, sugar and saturated fat; thus, we considered that any intake of these unrecommended food products would not increase DD [37]. Nevertheless, we performed an additional analysis including red meat in the score, as it has been considered previously [34], and there was no change in the results (data not shown).

Nevertheless, despite some limitations, there are several strengths of this study: (i) the inclusion of a representative sample of PCa cases, with specific information about aggressiveness, collected through pathological anatomy reports; (ii) the use of a DDS, that provides a more intuitive view of the whole dietary pattern, along with the variety of each of the food groups that have been addressed by the literature as important components linked with developing PCa; (iii) we included a considerable amount of baseline information collected using a standardized protocol that reduces information bias regarding reported food intakes, sociodemographic characteristics and lifestyles; (iv) most of the participants of the CAPLIFE study had detailed information about diet and credible energy intake which allowed the construction of DDS for 90.3% of PCa cases and 91.2% of controls; and (v) finally, to our knowledge, this is the first study to assess the association between DD and adequate micronutrient intake and PCa risk.

## 5. Conclusions

In this Spanish population-based case-control study, neither the DDS, the variety within food groups, nor the adequate nutrient intake were associated with PCa. It is important to report the negative results that we have observed for consideration in future research. Further studies with a prospective design and with an extended follow-up are needed to improve the knowledge of the relationship between a high-food diversity intake, adequate nutritional dietary content, and PCa.

## Figures and Tables

**Table 1:** Food groups and the recommended servings per day/week used in the dietary diversity score (DDS) according to the Spanish guidelines [25].

Food Groups	Food Subgroups	Recommended Servings
Vegetables	(1) Green vegetables: spinach, cruciferous, lettuce, green beans, eggplant, peppers, asparagus (2) Tomatoes (3) Yellow vegetables: carrots, pumpkin (4) Mushrooms	2 servings/day
Fruits	(1) Citrus fruits: orange (2) Tropical Fruits: banana, kiwi, grapes (3) Other seasonal fruits: Apple, peach, strawberries, watermelon, melon	3 servings/day
Dairy Products	(1) Milk: low fat and high fat (2) Yogurt: low fat and high fat (3) Cheese: low fat and high fat	2 servings/day
Cereals	(1) Potatoes (2) Grain: bread, pasta, rice, and whole breakfast cereals	4 servings/day
Protein food groups	(1) Legumes: peas, beans, lentils, and chickpeas (2) White meats: poultry and rabbit (3) Fish: oily fish, white fish and other shellfish/seafood (4) Eggs (5) Nuts: almonds, pistachios and walnuts	3 servings/week

**Table 2:** Characteristics of the controls and PCa cases in the CAPLIFE study.

	Controls		PCa Cases		*p* Value
	*n* = 302		*n* = 402		
Age (years), mean, (SD)	65.3	(8.2)	67.8	(7.4)	<0.001
BMI, mean, (SD)	28.7	(4.2)	28.5	(4.1)	0.533
BMI, *n*, (%)					
Normal weight (<25 kg/m^2^)	48	(15.9)	76	(18.9)	0.569
Overweight (25–29.9 kg/m^2^)	162	(53.6)	205	(51.0)	
Obesity (≥30 kg/m^2^)	92	(30.5)	121	(30.1)	
Marital status, *n*, (%)					
Married	254	(84.1)	338	(84.1)	0.938
Singled, never married	17	(5.6)	18	(4.5)	
Separated/divorced	20	(6.6)	27	(6.7)	
Widowed	11	(3.6)	19	(4.7)	
Education, *n*, (%)					
Primary	92	(30.5)	122	(30.4)	0.368
Secondary	147	(48.6)	212	(52.7)	
University	63	(20.9)	68	(16.9)	
Physical Activity (PA), *n*, (%)					
Low PA	113	(37.7)	157	(39.2)	0.350
Moderate PA	143	(47.7)	199	(49.8)	
High PA	46	(14.6)	46	(11.0)	
Smoking status, *n*, (%)					
Never	88	(29.1)	107	(26.6)	0.745
Former	157	(52.0)	214	(53.2)	
Current	57	(18.9)	81	(20.2)	
Alcohol consumption (g), mean, (SD)	11.6	(0.9)	14.1	(0.9)	0.046
Aggressiveness, *n*, (%)					
Low aggressiveness (ISUP 1–2)	-	-	309	(76.9)	-
High aggressiveness (ISUP 3–5)	-	-	93	(23.1)	-
First-degree family history of PCa, yes ^±^ *n*, (%)	15	(5.0)	25	(6.2)	0.478

Abbreviations: BMI: body mass index; PCa: Prostate cancer; SD: Standard Deviation; ± First-degree history of PCa: PCa history in father and/or brothers. Pearson chi-square test and Student’s t test were performed for evaluating differences in categorical and continuous variables, respectively.

**Table 3:** Assessment of the DDS and variety in each food group according to the tumor aggressiveness.

	Controls *n* = 302		PCa Cases *n* = 402			Low Aggressiveness *n* = 308			High Aggressiveness *n* = 93		
	Mean	(SD)	Mean	(SD)	*p* Value ^1^	Mean	(SD)	*p* Value ^1^	Mean	(SD)	*p* Value ^1^
Total DDS ^2^	5.5	(0.8)	5.6	(0.6)	0.217	5.6	(1.3)	0.399	5.8	(1.3)	0.165
Vegetables group ^3^	1.4	(0.6)	1.5	(0.6)	0.291	1.5	(0.6)	0.141	1.4	(0.7)	0.769
Fruits group ^3^	1.0	(0.6)	1.0	(0.6)	0.349	1.0	(0.6)	0.271	1.0	(0.6)	0.880
Cereals group ^3^	0.6	(0.4)	0.6	(0.3)	0.569	0.6	(0.4)	0.792	0.6	(0.3)	0.403
Dairy group ^3^	1.0	(0.6)	1.0	(0.6)	0.283	1.0	(0.6)	0.568	1.1	(0.6)	0.099
Protein group ^3^	1.6	(0.4)	1.6	(0.4)	0.396	1.6	(0.4)	0.662	1.7	(0.4)	0.282

Abbreviations: DDS: dietary diversity score; SD: standard deviation. Student’s t test was used in order to ascertain the differences between groups. ^1^ Controls as comparison group. ^2^ Total DDS ranged from a minimum of 0 points to a maximum of 10 points.^3^ Each food group ranged from a minimum of 0 points to a maximum of 2 points. One subject could not be categorized by aggressiveness as it was not an adenocarcinoma.

**Table 4:** Multivariable logistic regression models for the risk of PCa according to the total DDS and variety within the food groups.

	PCa Cases				
	cOR	95% CI	aOR ^a^	95% CI	*p* for Trend
Total DDS					
Q1 ^b^ (≥0.9, <4.7 points)	1 (ref.)		1 (ref.)		0.257
Q2 (≥4.7, <5.7 points)	1.07	(0.69, 1.66)	1.11	(0.69, 1.77)	
Q3 (≥5.7, <6.6 points)	1.61	(1.06, 2.46)	1.65	(1.05, 2.60)	
Q4 (≥6.6, <8.5 points)	1.18	(0.77, 1.83)	1.16	(0.72, 1.88)	
Vegetable group					
C1 ^c^ (0 point)	1 (ref.)		1 (ref.)		0.701
C2 (>0, <0.5 points)	1.29	(0.50, 1.83)	0.96	(0.49, 1.88)	
C3 (≥0.5, <1 point)	0.96	(0.70, 2.00)	0.97	(0.49, 1.89)	
C4 (≥1 point)	1.18	(0.71, 2.07)	1.08	(0.63, 1.87)	
Fruit group					
C1 (0 point)	1 (ref.)		1 (ref.)		0.159
C2 (>0, <1 point)	1.01	(0.62, 1.65)	0.90	(0.54, 1.50)	
C3 (≥1, <1.5 points)	0.64	(0.36, 1.11)	0.58	(0.32, 1.05)	
C4 (≥1.5 points)	0.91	(0.49, 1.71)	0.81	(0.42, 1.59)	
Cereal group					
C1 (0 point)	1 (ref.)		1 (ref.)		0.364
C2 (>0, <0.5 points)	1.38	(0.86, 2.21)	1.42	(0.88, 2.32)	
C3 (≥0.5, <1 point)	1.15	(0.67, 1.97)	1.18	(0.68, 2.06)	
C4 (≥1 point)	2.20	(0.86, 5.61)	2.31	(0.88, 6.03)	
Protein group					
C1 (0 point)	1 (ref.)		1 (ref.)		0.462
C2 (>0, <1 point)	1.26	(0.75, 2.10)	0.79	(0.44, 1.41)	
C3 (≥1, <1.5 points)	0.81	(0.46, 1.41)	0.82	(0.61, 1.39)	
C4 (≥1.5 points)	0.89	(0.60, 1.33)	0.92	(0.57, 1.24)	
Dairy group ^d^					
C1 (0 point)	1 (ref.)		1 (ref.)		0.336
C2 (>0, <1 point)	0.95	(0.59, 1.52)	0.93	(0.56, 1.52)	
C3 (≥1, <1.5 points)	1.37	(0.84, 2.24)	1.36	(0.81, 2.27)	
C4 (≥1.5 points)	1.05	(0.59, 1.88)	1.00	(0.55, 1.85)	

aOR: adjusted odds ratio; cOR: crude odds ratio. ^a^ Adjusted for age, BMI, educational level, physical activity level, smoking status, alcohol intake, and first-degree family history of PCa. ^b^ Q: Quartile, ^c^ C: Category, ^d^ Dairy group: low and high-fat milk, yogurt, and cheese.

**Table 5:** Prevalence of participants with an adequate, deficient, or excessive intake of nutrients according to 2/3 European Food Safety Agency (EFSA) Dietary Reference Intakes (DRIs) in the control and cases group stratified by the aggressiveness of the prostate cancer.

	Controls			PCa Cases							
	*n* = 302			*n* = 402							
				Low Aggressiveness (*n* = 308)				High Aggressiveness (*n* = 93)			
	DI ^a^	AI ^b^	EI ^c^	DI ^a^	AI ^b^	EI ^c^	*p* Value ^d^	DI^a^	AI^b^	EI^c^	*p* Value ^d^
Dietary Fiber (g/day)	7.0	93.0	-	7.5	92.5	-	0.806	8.6	91.4	-	0.594
Vitamin A (µg/day)	2.0	58.6	39.7	1.0	55.2	43.8	0.481	1.1	55.9	43.0	0.804
Vitamin B_6_ (mg/day)	0	100.0	0	0.6	99.4	0	0.170	0	100.0	0	-
Vitamin B_9_ (µg/day)	2.0	92.4	6.0	1.0	92.9	6.2	0.758	0	92.5	7.5	0.403
Vitamin B_12_ (µg/day)	0.3	99.7	0	0.3	99.7	0	0.964	1.1	99.0	0	0.386
Vitamin C (mg/day)	1.0	99.0	-	0.6	99.4	-	0.602	2.1	97.9	-	0.397
Vitamin D (µg/day)	66.6	33.4	0	70.8	29.2	0	0.261	57.0	43.0	0	0.092
Vitamin E (mg/day)	24.2	75.8	0	19.8	80.2	0	0.193	22.6	77.4	0	0.753
Calcium (mg/day)	4.0	85.1	10.9	3.3	86.7	10.1	0.829	2.2	85.0	12.9	0.634
Magnesium (mg/day)	0	0	100.0	0	0	100.0	-	1.1	0	98.9	0.071
Iodine (µg/day)	7.8	73.8	18.9	4.2	76.6	19.2	0.265	5.4	73.1	21.5	0.727
Potassium (mg/day)	0.7	99.3	0	1.0	99.0	0	0.669	1.1	98.9	0	0.688
Selenium (µg/day)	4.0	93.7	2.3	2.0	97.8	0.3	0.030	1.1	98.9	0	0.124

Intake (I): ^a^ deficient intake (DI), ^b^ adequate intake (AI), ^c^ excessive intake (EI). ^d^ Controls as comparison group. Values are % unless otherwise indicated. Pearson chi-square test was used in order to ascertain the differences between groups—there is no upper level intake (UL) in the micronutrient assessed.

**Table 6:** Logistic regression model for the risk of PCa and the micronutrient intakes of the dietary intake according to the recommendations proposed by the European Food Safety Agency.

Nutrient	PCa Cases				
	cOR	(95% CI)	aOR ^a^	(95% CI)	*p* for Trend
Dietary Fiber					
Adequate intake	1 (ref.)		1 (ref.)		
Inadequate intake ^b^	1.16	(0.65, 2.05)	1.34	(0.73, 2.44)	0.343
Vitamin A					
Adequate intake	1 (ref.)		1 (ref.)		
Inadequate intake	1.14	(0.84, 1.54)	1.07	(0.78, 1.47)	0.656
Vitamin B_9_					
Adequate intake	1 (ref.)		1 (ref.)		
Inadequate intake	0.94	(0.53, 1.67)	0.96	(0.53, 1.73)	0.886
Vitamin B_12_					
Adequate intake	1 (ref.)		1 (ref.)		
Inadequate intake	1.51	(0.14, 16.7)	1.68	(0.15, 19.4)	0.676
Vitamin C					
Adequate intake	1 (ref.)		1 (ref.)		
Inadequate intake	1.00	(0.22, 4.51)	0.91	(0.20, 4.19)	0.900
Vitamin D					
Adequate intake	1 (ref.)		1 (ref.)		
Inadequate intake	1.04	(0.76, 1.43)	1.11	(0.80, 1.55)	0.518
Vitamin E					
Adequate intake	1 (ref.)		1 (ref.)		
Inadequate intake	0.82	(0.57, 1.17)	0.86	(0.59, 1.25)	0.420
Calcium					
Adequate intake	1 (ref.)		1 (ref.)		
Inadequate intake	0.91	(0.59, 1.39)	0.95	(0.61, 1.48)	0.818
Iodine					
Adequate intake	1 (ref.)		1 (ref.)		
Inadequate intake	0.90	(0.64, 1.27)	0.91	(0.64, 1.30)	0.611
Potassium					
Adequate intake	1 (ref.)		1 (ref.)		
Inadequate intake	0.66	(0.12, 3.65)	0.67	(0.12, 3.84)	0.898
Selenium					
Adequate intake	1 (ref.)		1 (ref.)		
Inadequate intake	0.30	(0.13, 0.70)	0.33	(0.14, 0.79)	0.013

cOR: crude odds ratio; aOR: adjusted odds ratio; ^a^ Adjusted for age, smoking habits, physical activity level, educational level, alcohol intake, and first-degree family history of PCa. ^b^ Inadequate intake includes the category of deficient and/or excessive intake.
